# Light intensity and spectral distribution affect chytrid infection of cyanobacteria *via* modulation of host fitness

**DOI:** 10.1017/S0031182020000931

**Published:** 2020-09

**Authors:** Yile Tao, Justyna Wolinska, Franz Hölker, Ramsy Agha

**Affiliations:** 1 Leibniz-Institute of Freshwater Ecology and Inland Fisheries (IGB), Müggelseedamm 301, Berlin 12587, Germany; 2 Institute of Biology, Freie Universität Berlin, Königin-Luise-Straße 1-3, Berlin 14195, Germany

**Keywords:** Parasite traits, *Planktothrix*, *Rhyzophidum*, specificity, transmission, zoospores

## Abstract

Light gradients are an inherent feature in aquatic ecosystems and play a key role in shaping the biology of phytoplankton. Parasitism by chytrid fungi is gaining increasing attention as a major control agent of phytoplankton due to its previously overlooked ubiquity, and profound ecological and evolutionary consequences. Despite this interest, if and how light conditions modulate phytoplankton chytridiomycosis remains poorly studied. We investigated life-history traits of a chytrid parasite, *Rhizophydium megarrhizum*, under different light intensities and spectral compositions when infecting two closely related planktonic cyanobacteria with different light-harvesting strategies: *Planktothrix rubescens* and *P. agardhii*. In general, parasite transmission was highest under light conditions (both intensity and quality) that maximized growth rates for uninfected cyanobacteria. Chytrid encystment on hosts was significantly affected by light intensity and host strain identity. This likely resulted from higher irradiances stimulating the increased discharge of photosynthetic by-products, which drive parasite chemotaxis, and from strain-specific differences at the cell-surface. Comparisons of parasite transmission and host growth rates under different light conditions suggest the potential for epidemic development at higher irradiances, whereas host and parasite could coexist without epidemic outbreaks at lower light levels. These results illustrate the close relationship between parasite transmission and host fitness, which is ultimately modulated by the external environment.

## Introduction

The development and dynamics of disease are modulated by interdependency between the host, the parasite, and their environment. This relationship is captured by the ‘triangle of disease’ concept (Mcnew, 1960). Environmental variation can strongly modulate disease, affecting its severity and associated selective pressures (Wolinska and King, 2009) and, in some cases, can even lead to shifts from antagonistic to mutualistic interactions (Kiers *et al*., 2010*a*, 2010*b*). Besides more commonly studied environmental factors, such as temperature or nutrient limitation, quantitative and qualitative changes in light can be an important factor in determining the outcome of disease processes (Steinauer and Bonner, 2012; Johnson *et al*., 2016).

In aquatic ecosystems, primary production is driven mostly by phytoplankton. Phytoplankton communities in freshwater, brackish, and marine waters worldwide are often dominated by cyanobacteria. The excessive proliferation of cyanobacteria raises public health concerns due to the production of toxic metabolites by some taxa, and due to the severe disruption of ecosystem functioning (Havens *et al*., 2010). There is increasing evidence that cyanobacterial growth can be strongly regulated by biotic antagonists, such as parasites and pathogens (Gerphagnon *et al*., 2015). Among these, chytrids, i.e. fungi belonging to the phylum Chytridiomycota, display parasitic lifestyles and lethally infect cyanobacteria, diatoms, and other major phytoplankton groups (Frenken *et al*., 2017). Due to their inconspicuous morphology and difficulties in identification, chytrids have largely been neglected if not fully overlooked. Yet, interest in these fungi has grown in recent years, fuelled by environmental molecular surveys demonstrating their ubiquity and omnipresence in aquatic ecosystems (Hassett and Gradinger, 2016; Bochdansky *et al*., 2017; Ortiz-Alvarez *et al*., 2018; Hassett *et al*., 2020). Infection by chytrids is lethal and often reaches epidemic proportions, severely depleting phytoplankton populations, affecting phytoplankton succession, and causing delay or even complete suppression of bloom events (Davis *et al*., 2003; Frenken *et al*., 2017). Chytrids' most characteristic feature is a free-living life stage in the form of flagellated zoospores that actively seek suitable hosts by chemotaxis. Once encysted on their host, chytrids penetrate and extract nutrients from it, gradually developing into sporangia, chitinaceous reproductive structures that release new zoospores upon maturation (Ibelings *et al*., 2004). Chytrid parasites are increasingly considered to play important roles in the ecosystem. Besides their most evident effect on host abundances, succession patterns, and bloom formation, they also exert a strong selective pressure on phytoplankton that promotes genetic diversity in host populations (De Bruin *et al*., 2008; Gsell *et al*., 2013*a*; Agha *et al*., 2018*b*). Moreover, chytrid zoospores are effectively grazed by zooplankton consumers (Frenken *et al*., 2018), establishing a fungi-mediated trophic link that connects primary and secondary production in aquatic food webs (the so-called mycoloop, (Kagami *et al*., 2007)). Thereby, chytrid infection strongly contributes to the alleviation of trophic bottlenecks typically imposed by the dominance of poorly edible phytoplankton (Agha *et al*., 2016; Gerphagnon *et al*., 2019; Haraldsson *et al*., 2018; Frenken et al., 2020).

At present, our notions concerning the actual role of chytrids in aquatic ecosystems and the eco-evolutionary consequences of their infection are inferred, either from theoretical studies or from experimental observations in which conditions are lab-standardized and often not variable. Although it is expected that both host physiology and parasite transmission should be strongly modulated by field conditions, the effect of environmental variation on chytrid infection remains poorly characterized (but see (Frenken *et al*., 2017)). This will, in turn, modulate the extent and intensity of the eco-evolutionary feedbacks associated with chytridiomycosis (e.g. mycoloop and/or promotion of host genetic diversity). Environment-induced differences in host and parasite fitness determine infection dynamics, and can result in epidemics (when parasite transmission exceeds host growth rate), neutral host-parasite co-existence (i.e. host population growth exceeds that of parasites), or can establish environmental refuges from infection, where hosts can reside but parasites are unable to successfully infect or complete their life cycle. Pelagic ecosystems are characterized by strong vertical gradients in terms of temperature and light, which can strongly modulate chytrid infection severity. Thermal refuges from chytrid infection have been identified below 2°C and above 20°C for diatom hosts (Bruning, 1991*a*; Gsell *et al*., 2013*b*), and below 8°C for cyanobacteria hosts (Rohrlack *et al*., 2015; Agha *et al*., 2018*a*). Thermal refuges can be critical factors shaping seasonal phytoplankton phenology, as they provide an infection-free window (e.g. during early spring for diatom hosts), that can determine the intensity of subsequent blooms (Gsell *et al*., 2013*b*).

In addition to temperature, pelagic ecosystems display marked light gradients. As light passes through the water column, it is attenuated both in intensity and quality. Certain portions of the light spectrum are differentially scattered or absorbed depending on the colour of the water, the density of suspended particles, and the organic matter content. Given that chytrids rely on chemical cues to locate their host, and that such chemotaxis seems to be mediated by simple saccharides released as photosynthetic by-products (Scholz *et al*., 2017), light arguably has an important effect on the outcome of chytrid infection. Perhaps, in consequence, several chytrid taxa possess photoreceptors and show phototactic responses (Gleason and Lilje, 2009; Jekely, 2009). Chytrids have reduced mobility, and are unable to infect diatoms under dark conditions; reduced parasite infectivity and reproduction were observed at light intensities that were limiting for the host (Canter and Lund, 1951; Bruning, 1991*a*, 1991*b*, 1991*c*). It is unclear if these findings can be generalized to the infection of other phytoplankton groups. Particularly interesting in this regard are phytoplankton comprised of taxa that can inhabit depths with dissimilar light conditions, such as cyanobacteria. Different depth distribution among cyanobacteria species and/or conspecific strains results from polymorphism in intracellular gas vesicles that control cell buoyancy (Beard *et al*., 2000; Davis *et al*., 2003).

We evaluated the ability of a chytrid parasite to infect two cyanobacterial species of the filamentous genus *Planktothrix* under different light conditions. The selected species co-exist in nature and represent an illustrative example of niche partitioning. The discernable difference between the two species is their pigmentation. *P. rubescens* is a red, phycoerythrin-rich cyanobacterium, which typically inhabits deep, low-light metalimnetic waters. In turn, *P. agardhii* has a green-pigmentation, as it is rich in phycocyanin and chlorophyll, typically thrives in warmer epilimnetic waters where light is not limiting, and displays higher resistance to photoinhibition (Oberhaus *et al*., 2007). Otherwise, the species cannot be distinguished from one another morphologically, and genetic studies indicate that the two taxa are in fact conspecific (Humbert and Le Berre, 2001), a notion further supported by indications that genes encoding phycoerythrin in *P. rubescens* have been horizontally acquired (Tooming-Klunderud *et al*., 2013).

To explore the effects of light on the infection of cyanobacteria by chytrid fungi and to identify putative *Planktothrix* refuges from infection, we measured and compared host and parasite performance under different light intensities and spectral compositions. Specifically, we studied parasite traits related to various phases of infection, including (i) prevalence of infection; (ii) intensity of infection (i.e. mean number of individual infections per infected host), as a proxy of the ability of chytrid zoospores to locate and encyst on new hosts, and; (iii) the size of sporangia (i.e. parasite reproductive structures), as a proxy of the ability of the parasite to exploit its host, once encystment has taken place.

## Materials and methods

### Host and parasite strains

Two cyanobacterial strains of the filamentous, bloom-forming genus *Planktothrix* were used as hosts: NIVA-CYA98 (*Planktothrix rubescens*, isolated in 1982 from Lake Steinsfjörden, Norway) and NIVA-CYA630 (*Planktothrix agardhii*, isolated in 2008 from Lake Lyseren, Norway). The two lakes have been shown to be connected in terms of phytoplankton gene flow, and the strains are hence considered sympatric (Kyle *et al*., 2015). Host strains were routinely maintained as batch cultures in Z8 medium in a Binder KBW 720 incubator at 16°C (±0.1°C) under the continuous white light of 15 *μ*mol photons m^−2^ s^−1^. The chytrid parasite *Rhizophydium megarrhizum*, strain NIVA-Chy-Kol2008, was isolated in 2008 from Lake Kobotnvatet, Norway (Sonstebo and Rohrlack, 2011). This chytrid strain is capable of infecting both cyanobacterial strains (Agha *et al*., 2018*a*, 2018*b*). The parasite was maintained in culture by transferring chytrid zoospore suspensions into uninfected cultures of the host strain NIVA-CYA98 every 2–3 weeks as described in Agha *et al*. (2016).

### Experimental setup

Two independent experiments were conducted to assess the effect of light intensity and spectral composition on the parasite's ability to infect the cyanobacterial hosts. Host strains were acclimated as semicontinuous cultures to their respective light treatment by weekly diluting them back to an optical density at 750 nm of 0.05 (corresponding to the exponential growth phase) for at least 3 weeks prior to each experiment. Optical density at 750 nm showed a strong linear correlation with filament densities (Supplementary Fig. S1) and was used as a proxy of cyanobacterial biomass under uninfected conditions. Light intensity treatments were 0, 5, 10, 20 and 40 *μ*mol photons m^−2^ s^−1^ using a white cold fluorescent light (cyanobacteria in the dark treatment were acclimated at 5 *μ*mol photons m^−2^ s^−1^). Light quality treatments used were white, green, blue, or red light, all at an intensity of 20 *μ*mol photons m^−2^ s^−1^. Identical light intensities for these treatments were achieved by wrapping acclimation cultures and experimental flasks (50 mL sterile plastic tissue flasks) with transparent and/or coloured plastic foil layers. In order to measure the spectral composition and intensity of the incident light (same light source as in light intensity experiment) inside of the flasks, a compact spectrometer (Specbos 1211, JETI, Jena, Germany) was placed beneath mock flasks that were divided/cut in half longitudinally (the sensor did not fit inside intact flasks) and wrapped in transparent/colour foil ensuring the desired light spectrum for each treatment (Supplementary Fig. S2). Both infected and uninfected cyanobacterial cultures of each strain were included in the experiments, resulting in the following experimental design: Light intensity experiment: 2 cyanobacterial strains × 2 infection states (uninfected/infected) × 5 light intensities (0, 5, 10, 20, 40 *μ*mol photons m^−2^ s^−1^) × 6 replicates = 120 experimental units. Light quality experiment: 2 cyanobacterial strains × 2 infection states (uninfected/infected) × 4 light colours (White/Green/Blue/Red) × 4 replicates = 64 experimental units. Experimental units consisted of tissue flasks containing 30 mL of exponentially growing cyanobacteria at an optical density at 750 nm (OD_750nm_) of 0.05, measured with a Hach DR 3900 spectrophotometer. Infected experimental units were inoculated at the beginning of the experiment with purified suspensions of chytrid zoospores (final concentration 1600 and 7600 zoospores mL^−1^ for the light intensity and light quality experiments, respectively), obtained as described in Agha *et al*. (2018*a*). Briefly, a purified zoospore suspension was obtained by sequential filtration of a 10-day old infected culture through a sterile 5 *μ*m nylon-mesh and a 3 *μ*m polycarbonate filter (Whatman Nucleopore Track-Etch membrane). A measure of 1 ml of the filtrate was fixed with 0.1–0.2% of Lugol´s Iodine solution and zoospore density was determined using a Sedgewick Rafter counting chamber, under a Nikon Ti Eclypse inverted microscope.

Experimental units were maintained for 12 days under the relevant light conditions. For uninfected treatments, maximum cyanobacterial growth rates were calculated from daily measurements of OD_750nm_ using the R package ‘growthrates’ (Petzoldt, 2017). In previous experiments with this host-parasite system, OD_750nm_ showed a linear correlation with several biomass parameters, including filament density (Supplementary Fig. S1), biovolume and carbon content (data not shown), and was hence used here as a reliable proxy of cyanobacterial biomass under uninfected conditions (Agha *et al*., 2016, 2018*a*, 2018*b*). For the infected treatments, 2-mL samples were taken on days 4, 8 and 12, fixed in 2% formaldehyde, and used to determine the prevalence of infection (i.e. proportion of infected filaments, after inspection of 200 random filaments). In addition, for treatments resulting in enough infected filaments, intensity of infection (i.e. the mean number of sporangia present on single infected filaments, after inspection of 200 infected filaments) and size of mature/empty sporangia (at least 20) were determined. This was done for samples on day 12 only, in order to ensure a well-established level of infection. Sporangial size was determined by measuring their two semi-axes and treating them as rotational ellipsoids with volume
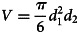
where *d*_1_ and *d*_2_ are the short and long semi-axes, respectively. If multiple mature/empty sporangia were present on the same host, only the biggest was measured. Parasite transmission rates were obtained from infection prevalence data by logistic regression, accomplished by fitting generalized linear models using a binomial distribution with a logit link function for each biological replicate (Agha *et al*., 2018*a*).

### Statistical analyses

Fixed and interactive effects of host strain and light intensity (or light quality) on each endpoint (i.e. parasite transmission rate, intensity of infection, sporangial size, host growth rate) were assessed by fitting linear models. Significant differences between treatments were identified by subsequent Tukey HSD *post hoc* tests. Data on intensity of infection and sporangial size could not be determined for samples where minimal or no infection was detected. The proportion of variance explained was calculated as the sum of squares of each term divided by the total sum of squares in the model. All statistical analyses were performed using RStudio (v.1.2.1335).

## Results

### Light intensity experiment

**Fig. 1.**
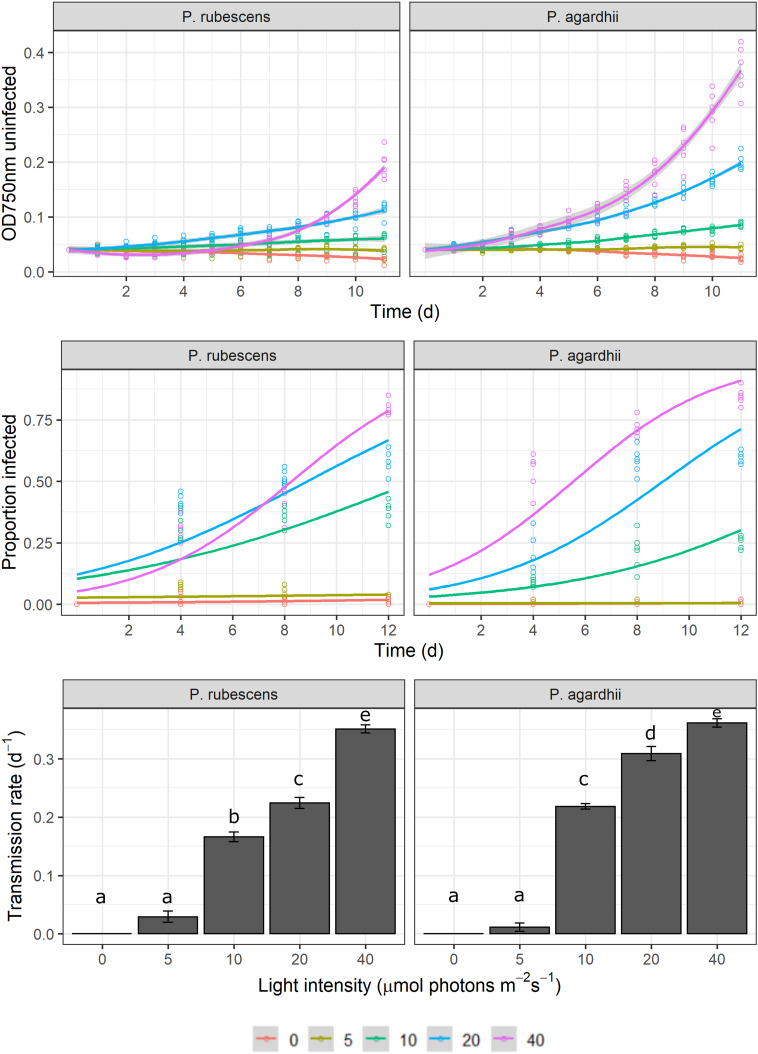
Change in cyanobacterial biomass (measured as optical density at 750 nm (upper panel), change in prevalence of infection over time (middle panel) and parasite transmission rates (lower panel) for the different light intensity treatments and host strains (*P. rubescens/P. agardhii*). The legend indicates the light intensity in *μ*mol photons m^−2^ s^−1^. Lines represent logistic fits of data, pooling all six biological replicates. Error bars depict s.e. Letters depict significant differences in parasite transmission rates (Tukey HSD test).

**Fig. 2.**
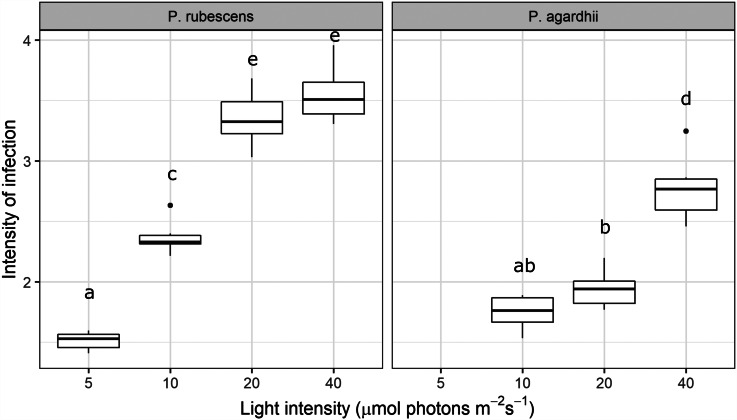
Intensity of infection recorded from every light intensity treatment and host strain. Missing boxplots result from marginal infection prevalence, which made it impossible to locate enough infected filaments to determine infection intensity reliably. Letters indicate significant differences (Tukey HSD test).

**Fig. 3.**
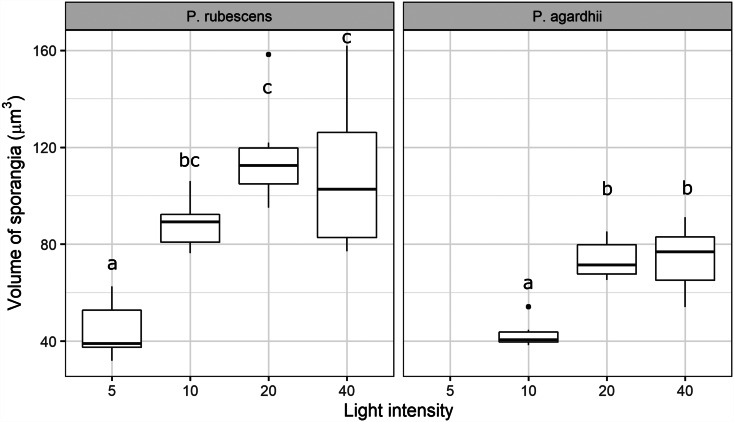
Sporangial sizes recorded for every light intensity treatment and host strain. Missing boxplots are due to marginal infection prevalence at the given light intensity, which made it impossible to locate enough filaments to determine sporangial size reliably. Letters indicate significant differences (Tukey HSD test).

**Fig. 4.**
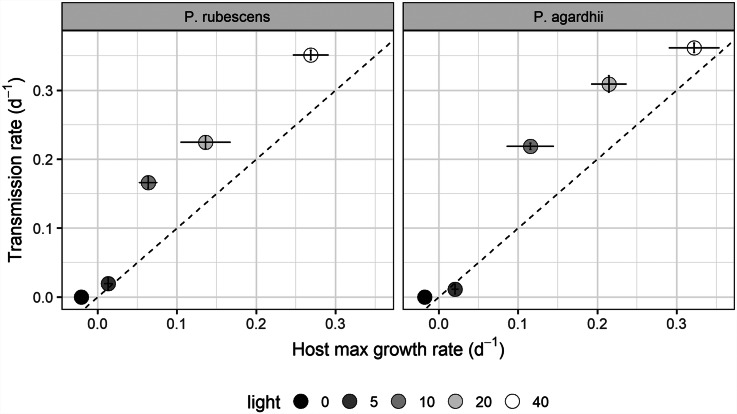
Parasite transmission rate plotted against the host growth rate for each tested light intensity. The dotted line indicates the boundary between epidemic development (above) and host-parasite coexistence (below). Error bars depict s.e.

Light intensity explained most of the variation in parasite transmission, intensity of infection, and sporangia size (96%, 91%, 80% of the variance explained, respectively; Table 1). Parasite transmission increased with light intensity, with no infection spread observed under dark conditions (Fig. 1). At intermediate light intensities (10 and 20 *μ*mol photons m^−2^ s^−1^) parasite transmission was higher in *P. agardhii*, whereas at low light intensities (5 *μ*mol photons m^−2^ s^−1^) parasite transmission was higher when infecting *P. rubescens* (see significant light intensity × host strain interaction; Table 1). Intensity of infection (i.e. the mean number of sporangia present on single infected host filaments) also increased with light intensity, and *P. rubescens* exhibited generally higher intensities of infection than *P. agardhii* (Fig. 2, Table 1). The size of chytrid sporangia was significantly affected by light intensity, with the two highest light intensities yielding the largest sporangia; host strain had no significant effect on this parameter (Fig. 3; Table 1). Uninfected cyanobacteria displayed higher growth rates with increasing light intensity, with *P. agardhii* consistently displaying higher growth rates than *P. rubescens* at moderate and high light intensities (⩾10 *μ*mol photons m^−2^ s^−1^; Table 1). No growth was observed under dark conditions, irrespective of host species. In order to predict whether infection can reach epidemic proportions under different light intensities or, conversely, allow for host and parasite co-existence, maximal growth rates of uninfected hosts and transmission rates of the parasite were plotted and compared for each tested condition (Fig. 4). Chytrid transmission generally exceeded host growth rates, indicating potential for epidemic outbreaks. Yet, at intensities ⩽10 *μ*mol photons m^−2^ s^−1^, parasite transmission rates were comparable to host growth rates, suggesting stable co-existence of host and parasite, and indicating that limited light conditions might prevent epidemic development.

**Table 1.** Linear models for fixed effects of light intensity, host strain and their interaction, on parasite transmission, intensity of infection, sporangial size and host growth rates

Light intensity experiment	df	*F* ratio	*P*-value	Variance explained (%)
Parasite transmission (*ß*)				
Light intensity	4	852.811	**<0.0001**	96.0
Host strain	1	29.773	**<0.0001**	0.8
Light intensity × Host strain	4	15.499	**<0.0001**	1.7
Residuals	50			1.4
Intensity of infection				
Light intensity	4	223.780	**<0.0001**	90.7
Host strain	1	19.714	**0.0006**	2
Light intensity × Host strain	2	15.772	**<0.0001**	3.2
Residuals	40			4.1
Sporangial size^a^				
Light intensity	4	45.109	**<0.0001**	80.1
Strain	1	1.14	0.29	0.5
Light intensity × Host strain	2	1.83	0.17	1.6
Residuals	40			17.8
Host growth rate (*μ)*				
Light intensity	4	89.1822	**<0.0001**	54.0
Host strain	1	14.9731	**0.0003**	22.7
Light intensity × Host strain	4	2.6082	**0.0465**	15.8
Residuals	50			7.6

df, degrees of freedom.

a Log-transformed.

Significant *P* values are depicted in bold. Proportion of variance explained is calculated as sum squares quotients.

### Light quality experiment

**Fig. 5.**
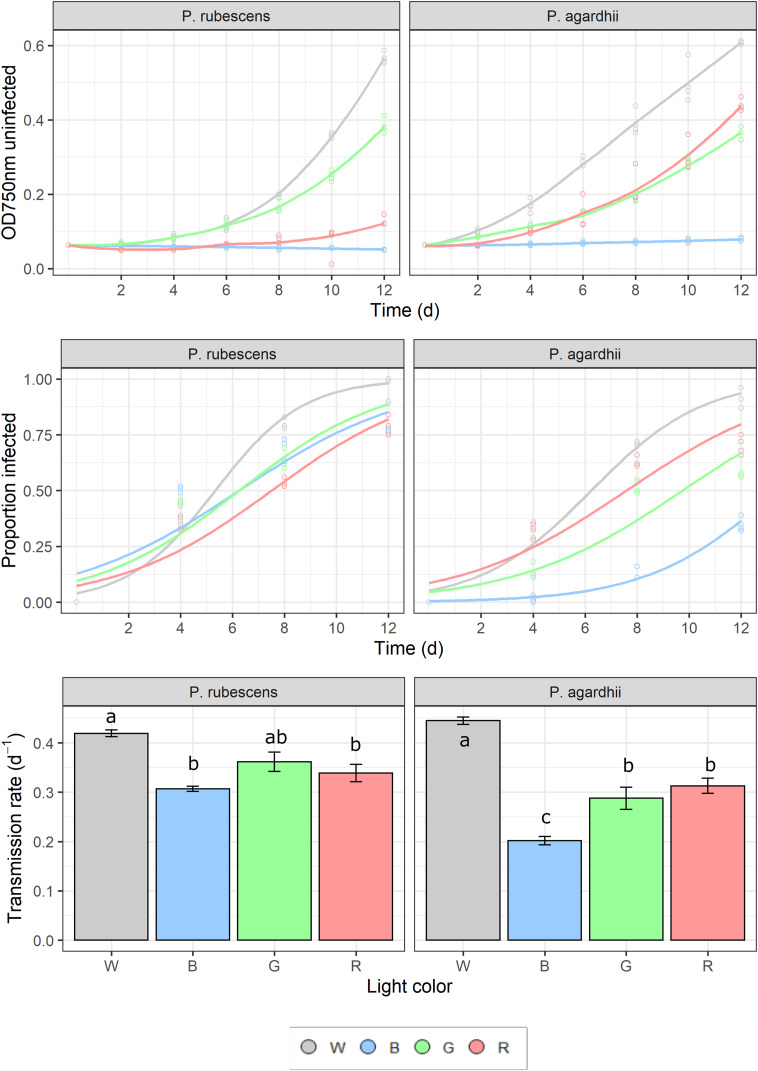
Change in cyanobacterial biomass (measured as optical density at 750 nm (upper panel), prevalence of infection over time (middle panel) and parasite transmission rates (lower panel) for the different light quality treatments and host strains. Lines represent logistic fits of data pooling all four biological replicates. Capital letters are used as an abbreviation of the individual light quality treatments: White, Blue, Green and Red light, respectively. Error bars depict SE. Significant differences after *post hoc* tests (Turkey HSD) are depicted as letters on the respective bars.

**Fig. 6.**
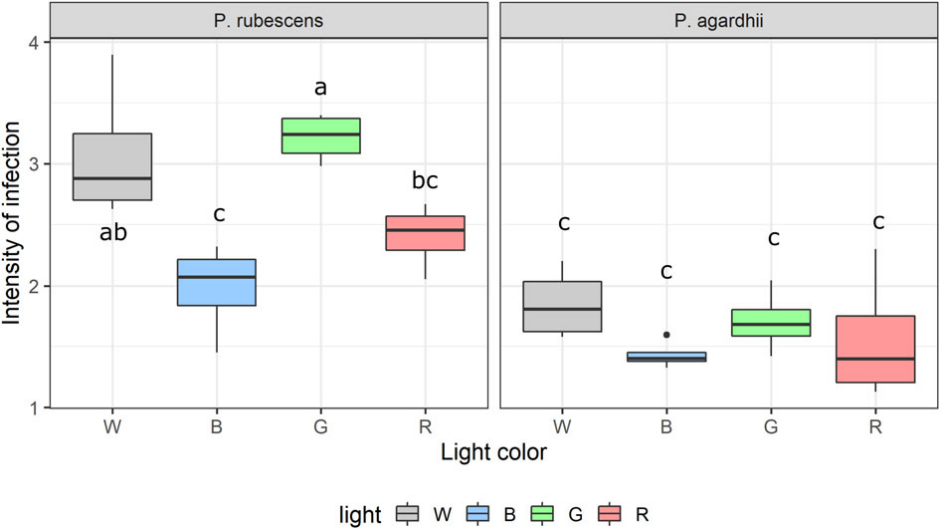
Intensity of infection (i.e. mean number of infections of host) recorded from every light quality treatment and host strain. Letters indicate significant differences for multiple comparisons (Tukey test).

**Fig. 7.**
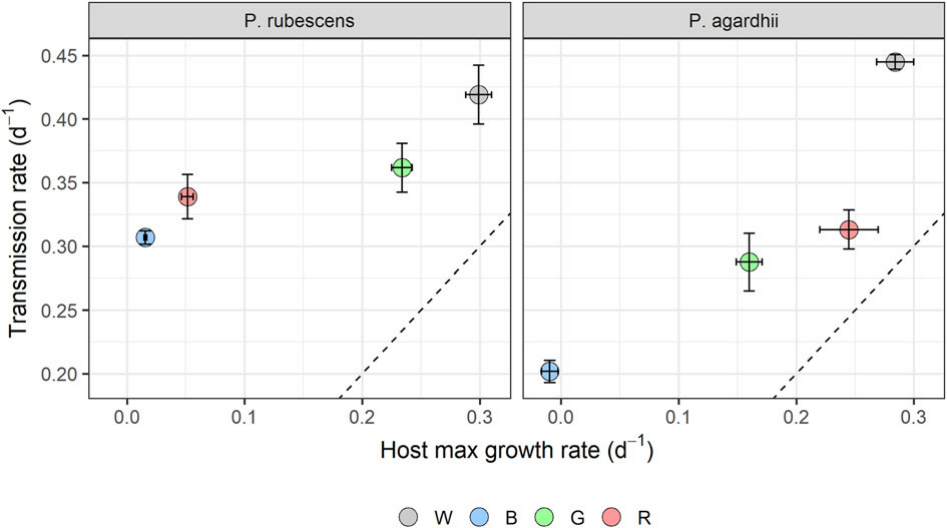
Parasite transmission rate plotted against the host growth rate for each tested light quality treatment. Data points over the dotted line indicate epidemic development (parasite transmission > host growth). Error bars depict s.e.

Light spectral distribution significantly affected parasite transmission and intensity of infection, but explained relatively less variance (72% and 18%, respectively) compared to light intensity. Parasite prevalence as well as parasite transmission were highest under white light for both host strains (Fig. 5). Blue light significantly suppressed parasite transmission in *P. agardhii,* but not in *P. rubescens,* compared to other light colours (see significant light quality × host strain interaction; Table 2). Light quality had a significant effect on the intensity of infection, but the effect size was small (18% of variance explained; Table 2). Highest intensities were recorded under white and green light, but only for *P. rubescens* (Fig. 6; see significant light quality × host strain interaction; Table 2). By contrast, intensity of infection was modulated more by host strain (53% of variance explained; Table 2), with *P. rubescens* consistently presenting higher intensities of infection than *P. agardhii*. Light colour did not significantly affect sporangial sizes (Supplementary Fig. S3). Both cyanobacterial strains showed the highest growth rates under white light and virtually no growth under blue light. Yet, *P. rubescens* showed comparatively higher growth rates under green light, whereas *P. agardhii* performed better under red light, reflecting their different pigment compositions and light harvesting strategies (Fig. 7). For both host strains, mean parasite transmission rates were higher than growth rates of uninfected hosts, independently of the light colour treatment, indicating that changes in light quality do not grant refuge from infection at homogenous light irradiances.

**Table 2.** Linear models for fixed effects of light colour, host strain and their interaction, on parasite transmission, intensity of infection, sporangial size and host growth rates

Light quality experiment	df	*F* ratio	*P*-value	Variance explained (%)
Parasite transmission (*ß*)				
Light colour	3	56.43	**<0.0001**	71.7
Host strain	1	4.755	**0.0393**	2.0
Light intensity × Host strain	3	12.345	**<0.0001**	16.1
Residuals	24			10.2
Intensity of infection				
Light colour	3	7.4208	**0.0011**	17.9
Host strain	1	65.9735	**<0.0001**	53.1
Light colour × Host strain	3	3.9978	**0.019**	9.7
Residuals	24			19.3
Sporangial size^a^				
Light colour	3	2.1636	0.1186	20.5
Host strain	1	0.0542	0.8179	0.2
Light colour × Host strain	3	0.3565	0.7849	3.4
Residuals	24			75.9
Host growth rate (*μ)*				
Light colour	3	183.606	**<0.0001**	77.2
Host strain	1	13.313	**0.001**	1.9
Light colour × Host strain	3	41.706	**<0.0001**	17.5
Residuals	24			3.4

df, degrees of freedom.

a Log-transformed.

Significant *P* values are depicted in bold. Proportion of variance explained is calculated as sum squares quotients.

## Discussion

Gradients of light intensity and quality in pelagic ecosystems delineate different habitats along the water column with effects on the physiology of phytoplankton hosts. Cyanobacteria display various strategies to maximize light utilization, including the production of accessory photosynthetic pigments, chromatic adaptation, or the use of intracellular gas vesicles to control their position in the water column (Carey *et al*., 2012). In this regard, the case of the genus *Planktothrix* is an illustrative example of niche partitioning, in which the two species display contrasting pigment compositions and inhabit different depths in the water column. *Planktothrix agardhii* inhabits epilimnetic waters characterized by high light intensity, high temperature, and low nutrient concentrations. In contrast, *P. rubescens* typically thrives in the metalimnion, where light is usually limiting and spectrally restricted to wavelengths in the green range, but where nutrient pulses from the underlying hypolimnion are more common. Differences in growth rates of *P. rubescens* and *P. agardhii* under green and red light in our experiment reflect their ability to maximize light utilization in their respective niches (Fig. 7).

How these different habitats modulate the pressure imposed by biological antagonists, such as chytrid parasites, remains unclear. In our experiment, light intensity had an overall positive effect on parasite transmission. Successful transmission results from the interplay of different parasite traits, including, but not limited to, the ability to locate and encyst on suitable hosts, and efficient exploitation of the host to produce parasite biomass (measured here as the intensity of infection and the size of sporangia, respectively; (Agha *et al*., 2018*a*)). Increased irradiance led in general to higher intensity of infection, supporting the notion that light is necessary for successful chytrid encystment on the host. This is consistent with reports of chytrid zoospores being strongly attracted to diatom colonies under light illumination, and quickly dispersing when irradiance was interrupted (Canter and Jaworski, 1980). Indications that zoospores locate their hosts through chemotaxis, with photosynthetic by-products (i.e. common carbohydrates) driving their attraction, further support this notion (Barr and Hickman, 1967*a*, 1967*b*; Scholz *et al*., 2017). Indeed, chytrid infection has been shown to be hampered in the presence of pollutants that inhibit algal photosynthesis (Van den Wyngaert *et al*., 2013). It seems that light intensity indirectly controls chytrid encystment *via* its effect on host photosynthesis, as supported by the pronounced effect of light intensity on parasite transmission observed in our experiment (Table 1).

In addition to light, differences among host strains had a significant effect on encystment compatibility. In another study testing the effect of temperature, the ability of a chytrid to encyst on the cyanobacterium *Planktothrix* was determined largely by host genetic background (Agha *et al*., 2018*b*). This was attributed to the fact that encystment is likely determined by interactions between hypervariable lectins-carbohydrates during cell-to-cell contact, as observed in other zoosporic fungi (e.g. Hinch and Clarke, 1980). This effect of host strain on chytrid encystment was also evident here, particularly in terms of differences in intensity of infection between host strains under identical light intensities. Still, light intensity appeared to be a stronger driver of chytrid encystment. By controlling host photosynthetic rates, light indirectly impacts chemotactic attraction of chytrid zoospores, a necessary process prior to parasite encystment and successful infection. Overall, chytrid encystment seems to be controlled in the first place by chemotaxis, which is itself driven by exudation of photosynthetic by-products (and therefore by light availability), and is further determined by parasite-host genotype compatibility at the cell surface level (i.e. cell–cell contact; Hinch and Clarke, 1980).

The size of mature or empty (i.e. fully developed) sporangia can be regarded as a proxy for host exploitation efficiency, that is, the efficiency with which the parasite utilizes host resources to produce its own biomass. Increased light irradiance led to bigger chytrid sporangia, likely as a result of higher host photosynthetic yields and associated higher intracellular resources, e.g. carbohydrates, available to the parasite. The light intensities used in our experiment are within the lower tolerance range of *Planktothrix*, and one could hypothesize that light intensities beyond this tolerance range (not tested here) might lead to host photoinhibition, reduce intracellular resources in the host and, thereby, negatively impact parasite fitness.

Light spectral distribution had a notable effect on infection outcome. Light preferences differ between *Planktothrix* strains in accordance with their dissimilar pigment compositions. Phycoerythrin-rich *P. rubescens* efficiently utilized green light, while *P. agardhii* showed maximum growth rates under red light, reflecting a Chl-a-based light harvesting strategy (Fig. 5; Oberhaus *et al*., 2007). Overall, parasite transmission was in line with such light preferences; more efficient light utilization by the host (as reflected by higher growth rates) led to higher parasite transmission, indicating that parasite fitness is closely related to the physiological state of the host. Similarly, higher intensity of infection coincided with light spectral distributions maximizing cyanobacterial growth, denoting, as for the light intensity experiment, a close relation between hosts´ photosynthesis and the ability of chytrids to encyst on them.

When comparing host growth rate and parasite transmission, it becomes evident that qualitative differences in light do not elicit marked changes in the outcome of infection. Instead, at low light irradiances, reduced parasite transmission rates closely matched host growth, indicating potential for host-parasite co-existence, without epidemic development. It is worth considering, however, that experimental conditions were optimal for parasite transmission. First, high host densities during incubations maximize host-parasite encounter rates. Under natural conditions, host densities are typically lower and, therefore, locating a suitable host for infection likely represents a bottleneck for parasite transmission. Second, the use of monoclonal strains in our experiment provides optimal, homogeneous host environments for the parasite and disregard the intraspecific diversity typically observed in natural phytoplankton populations (Rynearson and Armbrust, 2000; Rohrlack *et al*., 2008; Agha *et al*., 2014). Host diversity provides heterogeneous environments for the parasite, which slow parasite transmission and adaptation (Sonstebo and Rohrlack, 2011; Agha *et al*., 2018*b*). Given these considerations, we argue that parasite transmission rates might be inflated by experimental conditions and, therefore, the small differences between host growth and parasite transmission recorded at low light intensities could result in host populations growing faster than their chytrid parasites under natural conditions, preventing epidemic development. Therefore, low light intensities might represent a refuge from chytrid epidemics in the wild, allowing for host and parasite co-existence. A low light refuge from chytrid infection has been described for diatoms (Bruning, 1991*a*), which highlights the importance of light and photosynthetic output for the outcome of phytoplankton chytridiomycosis. The present results indicate the importance of the external environment affecting host, and thereby parasite, fitness, and determining the outcome of infection.

## Supplementary material

For supplementary material accompanying this paper visit https://doi.org/10.1017/S0031182020000931. click here to view supplementary material
