# Supplementary figures and images for: Light intensity and spectral distribution affect chytrid infection of cyanobacteria via modulation of host fitness
Source: Parasitology. 2020 Jun 9;147(11):1206–15. doi: 10.1017/S0031182020000931 (PMC7443748; doi:10.1017/S0031182020000931)

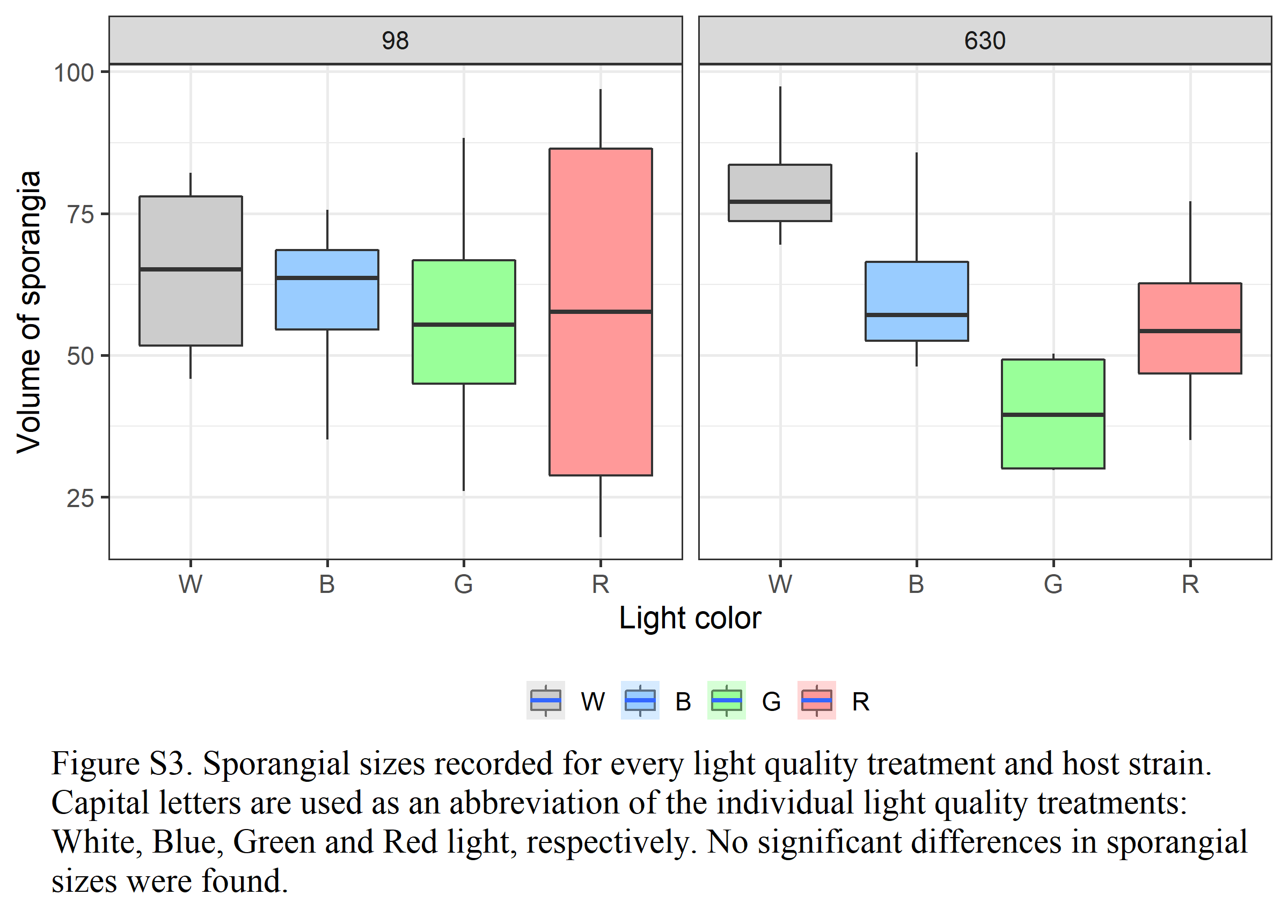

Supplement: Supplementary file 1 [file S0031182020000931sup.zip › S0031182020000931sup001.tif]

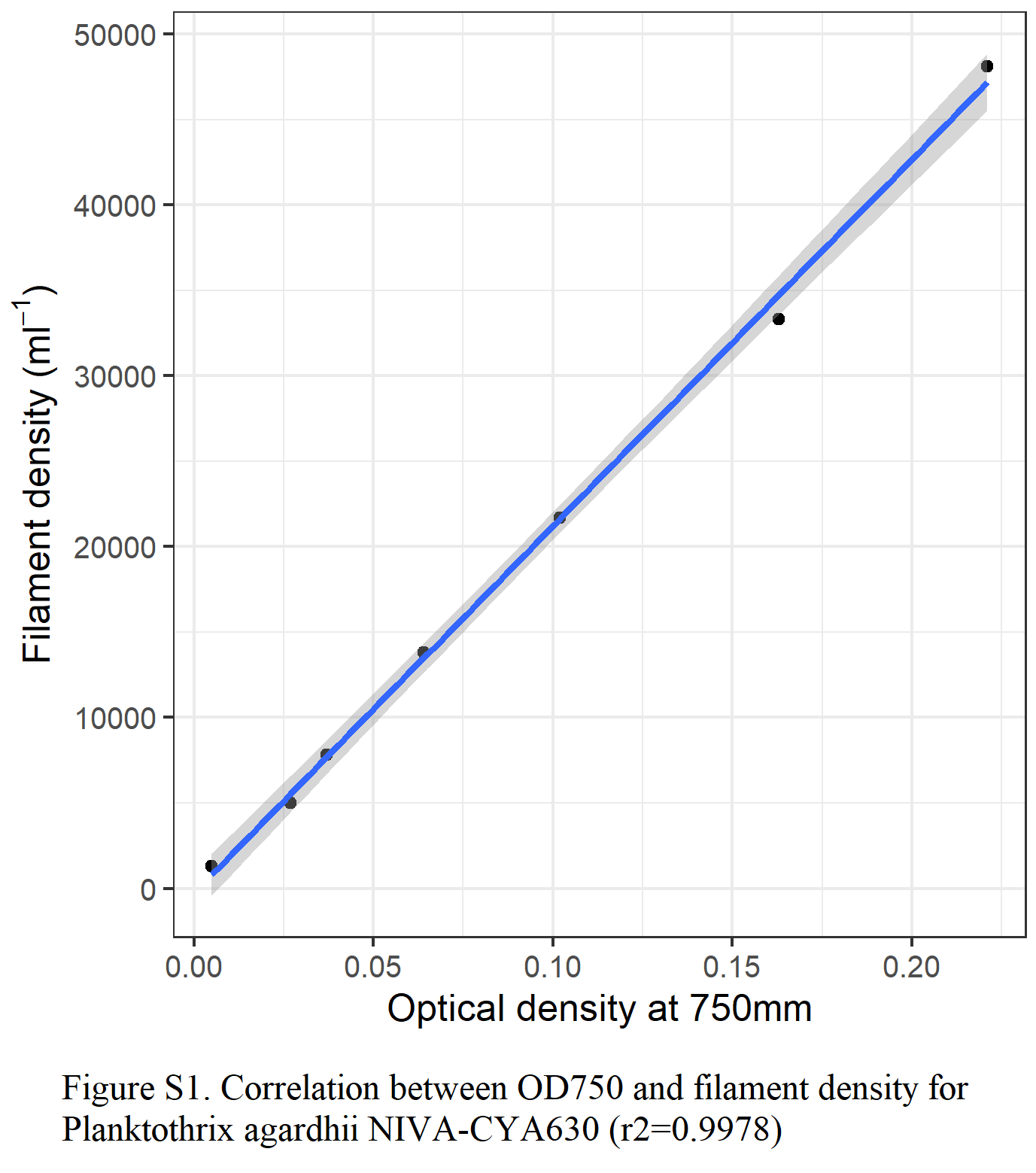

Supplement: Supplementary file 1 [file S0031182020000931sup.zip › S0031182020000931sup002.tif]

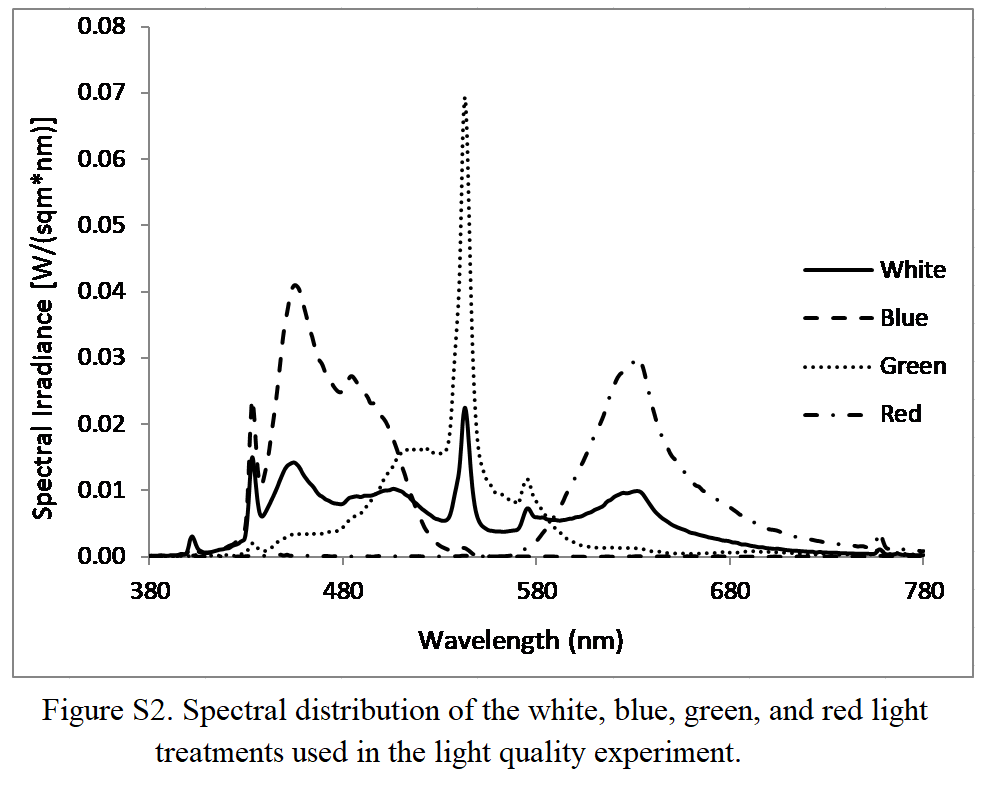

Supplement: Supplementary file 1 [file S0031182020000931sup.zip › S0031182020000931sup003.tif]
